# A novel analysis of gene array data: yeast cell cycle

**DOI:** 10.1093/biomethods/bpaa018

**Published:** 2020-09-04

**Authors:** Lawrence Sirovich

**Affiliations:** Center for Physics and Biology, Rockefeller University, New York, NY, USA

**Keywords:** cell division cycle, dynamic mode decomposition, *S. Cerevisiae* model

## Abstract

Many gene array studies of the yeast cell cycle have been performed (Cho RJ, Campbell MJ, Winzeler EA et al. A genome-wide transcriptional analysis of the mitotic cell cycle. Mol Cell 1998;2:65–73; Orlando DA, Lin CY, Bernard A et al. Global control of cell-cycle transcription by coupled CDK and network oscillators. Nature 2008;453:944–7; Pramila T, Wu W, Miles S et al. The Forkhead transcription factor Hcm1 regulates chromosome segregation genes and fills the S-phase gap in the transcriptional circuitry of the cell cycle. Genes Dev 2006;20:2266–78; Spellman PT, Sherlock G, Zhang MQ et al. Comprehensive identification of cell cycle–regulated genes of the yeast Saccharomyces cerevisiae by microarray hybridization. MBoC 1998;9:3273–97). Largely, these studies contain elements drawn from laboratory experiments. The present investigation determines cell division cycle (CDC) genes solely from the data (Orlando DA, Lin CY, Bernard A et al. Global control of cell-cycle transcription by coupled CDK and network oscillators. Nature 2008;453:944–7). It is shown by simple reasoning that the dynamics of the approximately 6000 yeast genes are described by an approximately six-dimensional space. This leads a precisely determined cell-cycle period, along with the quality and timing of the identified CDC genes. Convincing evidence for the role of the identified genes is obtained. While these show good agreement with standard CDC gene representatives (Orlando DA, Lin CY, Bernard A et al. Global control of cell-cycle transcription by coupled CDK and network oscillators. Nature 2008;453:944–7; Spellman PT, Sherlock G, Zhang MQ et al. Comprehensive identification of cell cycle–regulated genes of the yeast Saccharomyces cerevisiae by microarray hybridization. MBoC 1998;9:3273–97; de Lichtenberg U, Jensen LJ, Fausbøll A et al. Comparison of computational methods for the identification of cell cycle-regulated genes. Bioinformatics 2005;21:1164–71) several hundred newly revealed CDC genes appear, which merit attention. The present approach employs an adaptation of a method introduced to study turbulent flows (Schmid PJ. Dynamic mode decomposition of numerical and experimental data. J Fluid Mech 2010;656:5–28), “dynamic mode decomposition” (DMD). From this, one can infer that singular value decomposition, analysis of the data entangles the underlying (gene) dynamics implicit in the data; and that DMD produces the disentangling transformation. It is the assertion of this study that a new tool now exists for the analysis of the gene array signals, and in particular for investigating the yeast cell cycle.

## Introduction

This article shows that a rational analysis of yeast gene array data leads to an elementary model of the yeast life cycle. Simply stated, the yeast cell division cycle (CDC) can be viewed as an underdamped harmonic oscillator; and that each gene follows this dynamic with its own particular amplitude and phase.

Budding yeast, *Saccharomyces cerevisiae*, is a single cell eukaryote, perhaps the simplest of all. The cell contains a nucleus, the repository of DNA, and an assembly of organelles, e.g. endoplasmic reticulum, Golgi apparatus, ribosomes, etc. This content is typical of mammalian cells; thus the latter can be regarded as an extended version of the former. The yeast genome is composed of roughly 12 Mbp, compared to ∼3 Gbp found in mammalian cells; with roughly, 6000 yeast genes versus 21,000 human genes [1].

The blueprint of the yeast life form is contained in yeast’s 16 chromosomes [2]. The genome contains instructions for decoding itself, constructing itself, duplicating itself, and finally, inserting these commands into the constructed duplicate. This conforms to the overarching definition of an automaton, proposed by von Neumann, in a 1948 conference [3], well before the announcement by Watson and Crick of the double helix DNA model [4]. As Sydney Brenner, in [5] observed, a history of molecular biology might be written from the von Neumann perspective, but the coincidence of concepts is entirely retrospective.

Two theoretical ideas lie at the heart of the present construction. One is the Beadle and Tatum hypothesis “one gene-one enzyme” [6], later to become “one gene-one polypeptide.” The second is “DNA makes RNA makes protein,” referred to as the Central Dogma, and attributed to Frances Crick [7]. It too was subsequently recast more generally.

The fate of a budding yeast cell is mitosis division into two daughter cells, each conforming to the mother. The process of division contains two acts: Synthesis, S, and Mitosis, M; and two entr'actes: gap1, G1 and gap 2, G2, during which the motif changes. This play has a duration of at least an hour, and under proper conditions yeast cell division continues indefinitely, with population doubling each cycle: →G1→ S→ G2 →M→ G1.

During the course of the cell cycle, DNA and the full range of organelles are duplicated, through processes broadly dubbed as transcription and translation, which involve production of mRNA and other polypeptides that lead to the daughter copies of the mother cell. The dynamics of transcription and translation have time-scales ≈1 min [1], accompanied by additional, shorter, sub-events.

As a conceptual background to the present viewpoint, consider a volume of gas, with ∼10^23^ interacting molecules. The mean time between collisions, *t*, and the mean free path, l, characterize the internal state of the gas (Incidentally, l/t ≈ the speed of sound.). A coarse-grained description, for times ≫ *t* and spatial scales ≫ l, then leads to a satisfactory thermodynamic description of the gas, in terms of: density, *ρ*, and pressure, *p*; instead of the dynamics of 10^23^ interacting molecules [8].

If the yeast cell cycle is coarsely sampled, then over many minutes, “translation and transcription” and their sub-events are averaged out, and from this, it follows that only genes and their proteins figure in the description, i.e. the Beadle–Tatum view.

## Material and methods

### Yeast cell cycle data

At the end of the last century, micro-array studies appeared for yeast (*S. cerevisiae*), that used course-grain time sampling of gene expression levels, over the course of the cell cycle [9, 10]. These studies followed the roughly 6000 yeast genes over the cell cycle, by recording mRNA expression levels of the genes. To enable such data acquisition, yeast populations were assembled by various means so as to contain an initial homogeneous population of cells. For example, by elutriation, a population of newly minted daughter cells could be extracted.

Attention will be confined to the Orlando *et al.* [11] database, henceforth referred to as (I). Their study followed the expression levels of 5716 genes of *S. cerevisiae* for the wild-type (WT), and also for a mutant strain. WT data will mainly be considered here. As might be expected from gene array data, noise is a factor. However, in acquiring repeated databases, these authors provide convincing evidence of reproducibility.

### The database

Yeast populations of (I), composed largely of daughter cells, were sampled 15 times at 16 min intervals, consistent with temporal coarse graining. The matrix of gene expressions of the first WT dataset will be denoted by the array

(1) $${\;}_{5716}G{}^{15},$$

as indicated, *G* is composed of the 5716 sampled genes, as rows of the 15 sample times, at intervals

(2) dt=16 min.

The mean of each sequence is subtracted, $\sum_{j}G_{\text{ij}}=0,$ and we define,

(3) $$Z=G-\bar{G;}\;\bar{\;G}={\langle G\rangle }_{t}.$$

While 5716 genes may appear daunting the true dimension is 15. Treatment of this database falls under the “method of snapshots” [12] which demonstrates that the analyses of any database can be reduced to the minimal dimension of the data, 15 in the present instance. To carry out this calculation, the 15 × 15 symmetric nonnegative matrix, $Z^{†}Z$ is formed, and an Eigen analysis is applied,

(4) $$Z^{†}ZV=V\Lambda .$$

*Λ* is the diagonal matrix of eigenvalues, λ_j_, arranged in descending order of magnitude. The columns of the eigenvector matrix, *V*, correspond to the associated time courses. Any gene expression can be represented as an admixture of these 15 columns.

The matrix, *Z*, Equation (1), has the singular value decomposition (SVD) representation [13] (see [14] for an SVD analysis of the [10] database),

(5) $$Z=U\Lambda V^{†}=\sum_{j=1}^{15}u_{j}\sigma_{j}v_{j}^{†};\;\sigma_{j}=\sqrt{\lambda_{j}},$$

where {*v_j_*}, are the columns of *V*. The terms of Equation (5) are ordered in decreasing size of $\lambda_{j}.$ The column vectors, *U*, of length 5716 are the eigenvectors of $ZZ^{†},$ but are more easily obtained from the columns of

(6) $$U=ZV\Lambda^{-1},$$

Both {*u_j_*} and {*v_j_*}, as eigenvectors of symmetric matrices, each form orthonormal sets. It is important to observe that Equation (5) formats the data in a factored form: gene features, *U*, and dynamics, $\Lambda V^{†}$. In what follows, discussion is simplified by the introduction of

(7) $$T=\Lambda V^{†}=\left[\begin{array}{ccc} \tau_{1} & \cdots & \tau_{N}\\ \vdots & \vdots & \vdots \end{array}\right],$$

which describes the dynamics.

We pause to compare the second WT database contained in (I). If we denote its eigenvector matrix by *V_2_*, then a suitable measure of reproducibility of the two WT databases is furnished by the 15 × 15 correlation matrix,

(8) $$V^{†}V_{2},$$

depicted below.

**Figure 1:**
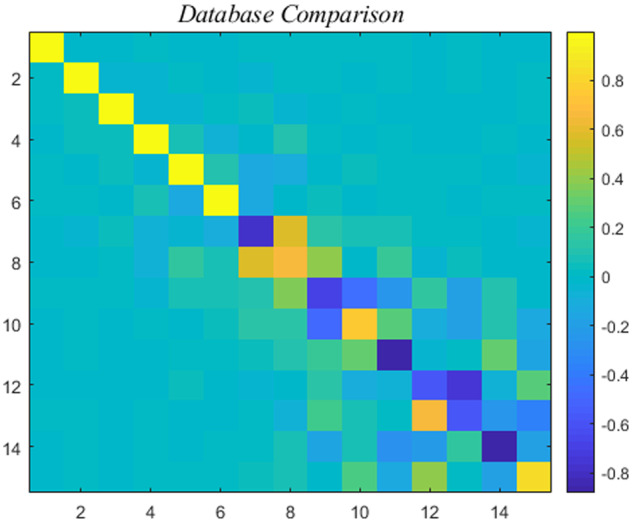
Correlation levels, see color bar, of the two WT databases obtained in (I).

Figure 1 shows high correlation for the first six subspaces. The remaining nine subspaces are considered noise. Further verification comes from the “energy” norm, i.e. the square of the Frobenius norm. As indicated by the name it denotes the energy in physical situations. Under this norm

(9) $${\left\|Z\right\|}^{2}{}_{F}=\sum_{i,j}Z_{i,j}^{2}=\sum_{k}^{15}\lambda_{j}.$$

**Figure 2:**
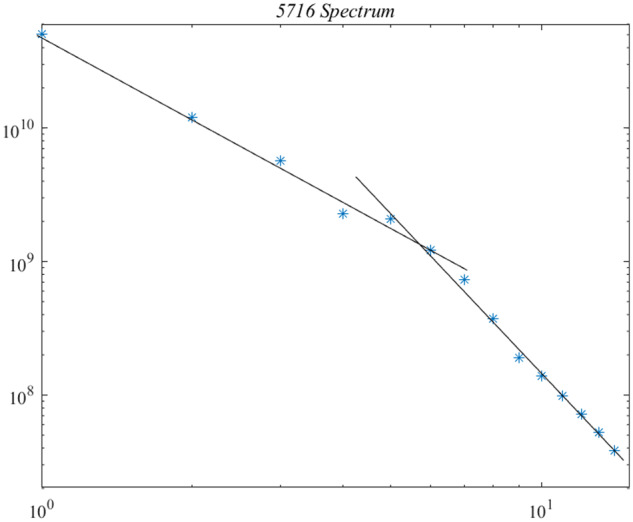
Log-log plot of the 15 eigenvalues of Λ, arranged in decreasing magnitude. The presence of a *knee*, or crossing point, is generally regarded as a transition from signal to noise.

A direct calculation reveals that

(10) $$\sum_{7}^{15}\lambda_{j}/\sum_{1}^{15}\lambda_{j}=O(10^{-6}),$$

which confirms that the last nine subspaces represent noise. Another perspective, is furnished by the log–log plot of eigenvalues, shown below in Fig. 2. The eigenvalues are clearly well fit by two straight lines indicating two different power-law descriptions. In a time-honored tradition, the left collection is associated with signal, large *λ*, and the right with noise, small *λ.* Under this hypothesis, the signal is contained in the first six eigenmodes.

The agreement of Figs. 1 and 2 confirms the quality of the data. Henceforth, attention is restricted to the first six modes,

(11) $$X=U_{r}T_{r}=\sum_{j=1}^{6}u_{j}\tau_{j}^{†},$$

where *u* is a 5716 element column vector, and$\tau^{†}$ is a 15 element row vector.

(12) $${\left(U_{r}\right)}_{i,j}=U_{i,j},j=1,..,6,$$

and similarly *T_r_,* is 6 × 15. Equation (11) is an “example” of a low-rank approximation [15], and SVD has the property of being the best *N*^th^ order approximation to *X,* for any *N*, the Schmidt–Eckart–Young–Mirsky theorem [16].

### Dynamic mode decomposition

**Figure 3:**
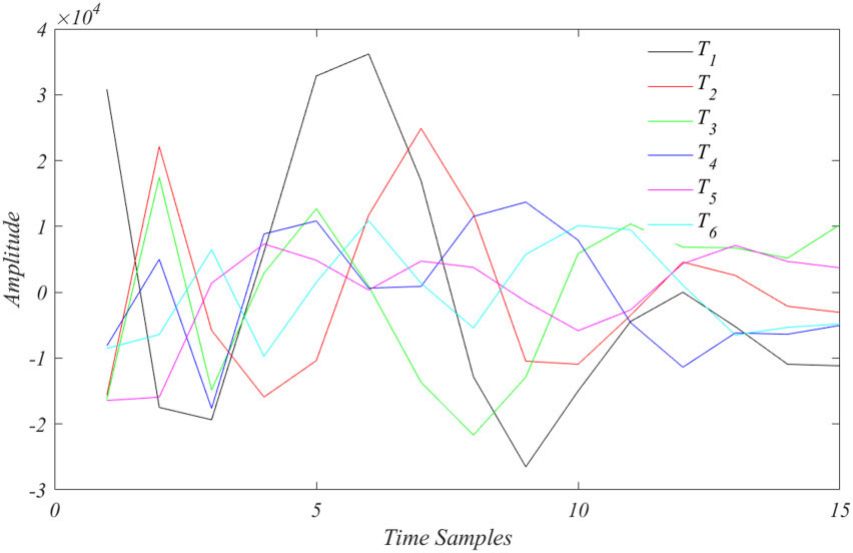
The temporal behavior of the top six SVD modes, Equation (11), *T_r_*.

The six SVD temporal modes of *V* are depicted in Fig. 3. From the experiment giving rise to the analyzed data, one might reasonably suppose that exponential decay and sinusoidal expression are main events. However, what we see in Fig. 3 appears to be a hodgepodge of behaviors, some passably sinusoidal, some passably exponential. The anticipated behavior appears to be “entangled,” an abiding shortcoming of SVD. As emphasized above SVD is a mathematically optimal representation of the data, but of uncertain scientific interpretation for the variables, *U* and *V*. This section shows how the data can be “disentangled,” by a procedure referred to as dynamic mode decomposition (DMD).

Focus will be on the near periodic phenomena of cell division, and the identification of genes mobilized to carry out the CDC. Help comes from an analytic framework for treating turbulent fluid dynamics [17], which produces a framework for disentangling multimodal phenomena, dubbed DMD. A monograph on DMD [18], displays the rich range of phenomena to which DMD may be applied. For earlier references, and especially the program proposed by [19] see [17, 18]. Appendix section contains an outline of the basic DMD concepts adapted to the present situation.

In brief, consider Equation (11), which in column format can be written as

(13) $$X=\left[\begin{array}{ccc} x_{1} & \cdots & x_{N}\\ ↓ & \cdots & ↓ \end{array}\right],$$

where *N* = 15. As an overall concept, under DMD, a constant matrix, *A* is sought, such that

(14) $$\sum_{k=1}^{N-1}{\left\|x_{k+1}-Ax_{k}\right\|}^{2},$$

is minimized. This translates into the determination a “useful” linear approximation of the dynamics so that

(15) $$x_{k+1}\approx Ax_{k}.$$

As shown in the Appendix, Equation (14), the search for *A* can be reduced to consideration of a 6 × 6 matrix, $\;\tilde{A}\;,$followed by its Eigen-decomposition, $\tilde{A}={\text{WDW}}^{-1}.$  *D* is the diagonal matrix of eigenvalues, *µ_j_, j* = 1, 0.6, is displayed on the first line of Table 1 below. Under this formulation, *T* takes on the form, Equation (15),

(16) $$T=W\left[D^{0},D^{2},\ldots ,D^{14}\right]\Phi_{0}=W\Phi ,\;\;\Phi_{0}=W^{-1}T_{1}.$$

**Table 1:** the six *µ* and *Ω* values

µ	−0.4433	0.3182 + 0.8105i	0.3182–0.8105i	0.5376 + 0.2992i	0.5376–0.2992i	0.3953
Ω	NA	−0.0087 + 0748i	−0.0087–0748i	−0.0304 + 0.0317i	−0.0304–0.0317i	−0.0580

To extend the discrete form, Equation (16), to an exponential (continuous) form, define

(17) $$\Omega_{j}=\frac{\;\text{log}(\mu_{j})}{dt},$$

The Ω values are displayed on the second line of Table 1 below. There is no continuous version of the first entry of µ, which contains an artifact of sampling.

For the data (I), as given in the form Equation (13), the eigenvalues of *D* are shown in Table 1. Note that these are real or occur as conjugate pairs, a reflection of the fact that all analyses should render real-valued results.

**Figure 4:**
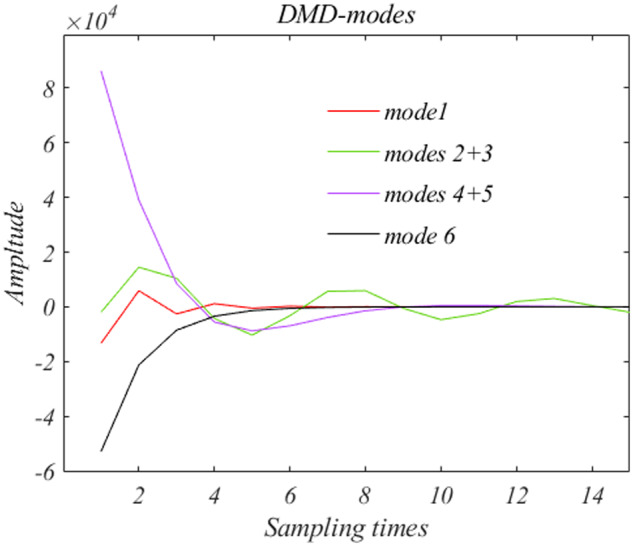
The time courses of the six modes are displayed. Modes 2 and 3 are conjugate, as are 4 and 5.

Comparison of Fig. 4 with Fig. 3 demonstrates that the goal of rendering the dynamics into individual component modes has been accomplished.

The corresponding dynamical modes, >15 samplings are displayed in Fig. 4. All modes show some decay. The initial population of yeast cells, obtained through elutriation, produces a stressed nonequilibrium, and exponential return to some form of equilibrium should be expected. The oscillation due to Modes 2 and 3 can reasonably be regarded as describing cell division. Exponential decay is due to variability in cycle time of daughter cells. Modes 2 and 3 will be the focus in the following deliberations.

## Results

### Yeast cell cycle

The DMD representation of the yeast data *X*, Equation (11), as developed in the Appendix is given by

(18) $$X=U_{W}\Phi ,$$

where *U_W_=U × W* is the 5716 × 6 matrix, of gene weightings, and Φare the six time courses exhibited in Equation (16). In the interest of clarity, we express Equation (18) as the following decomposition

(19) $$U_{W}\Phi =U_{W1}\Phi_{1}+U_{W2}\Phi_{2}+U_{W3}\Phi_{3}+\cdots +U_{W6}\Phi_{6}\approx U_{W2}\Phi_{2}+U_{W3}\Phi_{3}=X_{\text{CC}}.$$

Since $X_{\text{CC}}$ is real, it’s two terms are conjugates of each other,. If this is transformed to the continuous version, Equation (17), then

(20) $$\Omega_{2}=-0.0087+0.0748i=\Omega_{r}+i\Omega_{i},$$

and $\Omega_{3},$the conjugate. An immediate result, is the period of cell division,

(21) $$T_{\text{cc}}=\frac{2\pi }{\Omega_{i}}\approx 84\;\text{min},$$

an estimate of the cell-cycle period, free of modeling. (The second WT experiment, when subjected to the same analysis gave an estimate of *T_c_* ∼97.5 min.) In passing, note that the sampling frequency is roughly 0.2, and thus the Nyquist–Shannon criterion is satisfied [20], see the Appendix. Based on their expertise, the authors of (I) estimated the average cell cycle to be ∼95 min, based on a mother cell-cycle period of ∼77 min and a daughter cell cycle of ∼118 min; and that sampling times entered the third cycle (private communication, Steven Haase). Thus, agreement with the experimental observations might be regarded at least as passable.

Based on the above deliberations, the pair of cell cycle modes*, X*_cc_ can be expressed as,

(22) $$X_{cc}={\left(R\times e^{i\varphi }\right)}_{U}\times {\left(\rho e^{i\theta +\Omega t}\right)}_{\Phi }+c.c=2R\rho e^{\Omega_{r}t}\;\text{cos}(\Omega_{i}t+\varphi +\theta ),$$

where *R* and *φ* represent the magnitude and phase, respectively, of each of the 5716 gene expressions. The phase *φ,* is a surrogate for onset time of expression for the gene. As such, it provides the gene ordering of sequences. As above $\Omega =\Omega_{r}+i\;\Omega_{i}$. The subscripts *U* and *Φ* gene contribution and temporal dynamics, respectively. It follows from the data that

(23) ρ≈22332;  and  θ=1.7623,

the amplitude and phase for the dynamical mode.

Equation (22) is recognizable as the solution of an under-damped harmonic oscillator, governed by,

(24) $$\frac{d^{2}\eta }{dt^{2}}=2\zeta \omega \frac{d\eta }{dt}+\omega^{2}\eta ,$$

and solution ${\left(\rho e^{i\theta +\Omega t}\right)}_{\Phi },$in Equation (22). The frequency, *ω*, and dimensionless damping factor ζare given by

(25) $$\zeta =\Omega_{r}/\omega ∼.12\;\&\omega =\Omega_{i}/\sqrt{1-\zeta^{2}}.$$

The “true” frequency is a small correction to the above-calculated value.

**Figure 5:**
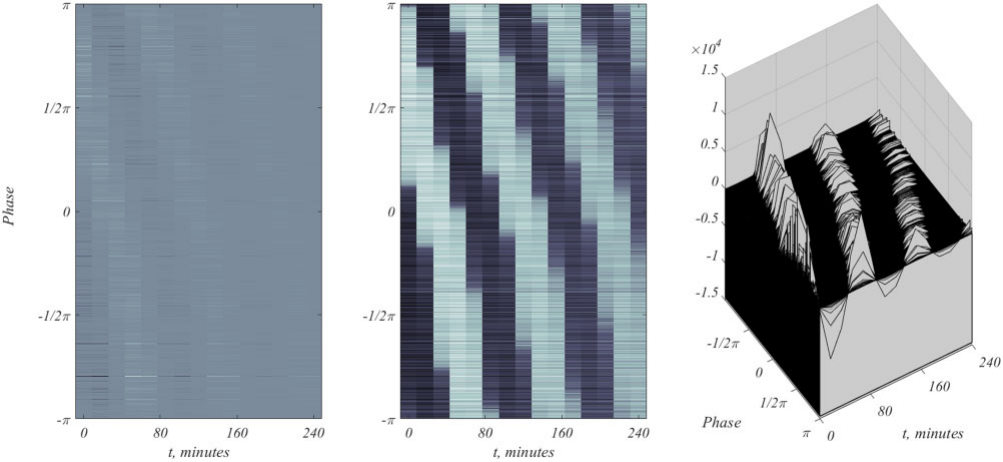
The figure on the left shows the relatively weak signal. To enhance the effect, the Figure has been subjected to a log transformation, middle figure. The jagged appearance is due to the 16-min sampling times. The middle panel of Fig. 5 is just an enhancement. The rightmost panel is the three-dimensional version of all 5716 time courses, which shows the paucity of sharp maxima, the presumed evidence for the CDC.

If the 5716 sequences are ordered in decreasing phase, *φ* [9–11, 21], then the 5716 × 15 matrix *X*_cc_ can be viewed as the image in the left panel of Fig. 5. According to Equation (22)  *φ* as a function of *t* should carry a negative slope, as faintly evidenced in Fig. 5, referred to as a phase wave.

The left most panel of Fig. 5 conveys a faint signal. Most genes respond with near-constant activity. It is estimated that roughly 8–12% of the genes participate in the CDC. The vast majority of genes does not participate, and might be deemed to be “housekeeping” genes, responsible for a steady supply of ingredients needed by a typical cell. We can consider the time of maximal gene expression, *t*_m_, as described by Equation (22), also see. From Equation (21), this is given by

(26) $$t_{m}/T_{\text{cc}}+\frac{\theta }{2\pi }=\frac{\varphi }{2\pi }\mathrm{mod}(1)$$

which is clearly proportional to *φ.* The expression level at this time is given by the amplitude

(27) $$M_{\text{cc}}=\max{\left(X_{\text{cc}}\right)}_{t}.$$

### Cell division genes

In [11] a collection of 440 cell-cycle genes, WTCON, are assembled based on commonality with related investigations [9, 10, 22,]. In this section, we obtain a full complement of CDC genes, based solely on the data itself. For this purpose, the total gene signal will be written in the approximate form,

(28) $$G_{\text{cc}}=\bar{G}+X_{\text{cc}}.$$

A criterion for distinguishing cell cycle versus housekeeping genes can be discussed in terms of

(29) $$C_{V}=\frac{M_{\text{cc}}}{\bar{G}},$$

a “coefficient of variation,” for each gene. If *C_V_* is relatively large, then it is a candidate CDC gene, and if *C_V_* is relatively small, it is a candidate housekeeping gene. As a nominal case we consider $C_{V}>0.475.$ There are approximately 400 such gene candidates, which we denote by WT400 (actual number is 403), that meet this condition, this number is roughly 8% of the total number of genes. The WTCON set only shares ∼30 with the presently proposed set of WT400 genes.

**Figure 6:**
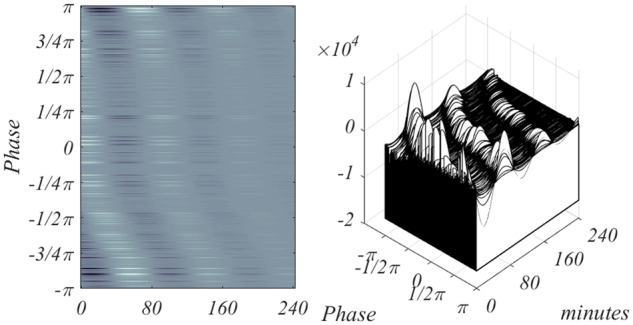
Depiction of the WT400 CDC genes. In both images time, measured in minutes, runs over more than two periods of the CDC. In both instances, the continuous model Equation (22) is used to generate the images.

To test the validity of WT400 set, restriction to these genes is considered, and the result is the left image of Fig. 6, which clearly shows the phase wave associated with CDC. The right panel shows all 407 time courses, a dense collection of peaking gene expressions. The criterion values of $C_{V}$, used to select the 407 sequences is nominal, and will be further considered.

### First proof of concept

**Figure 7:**
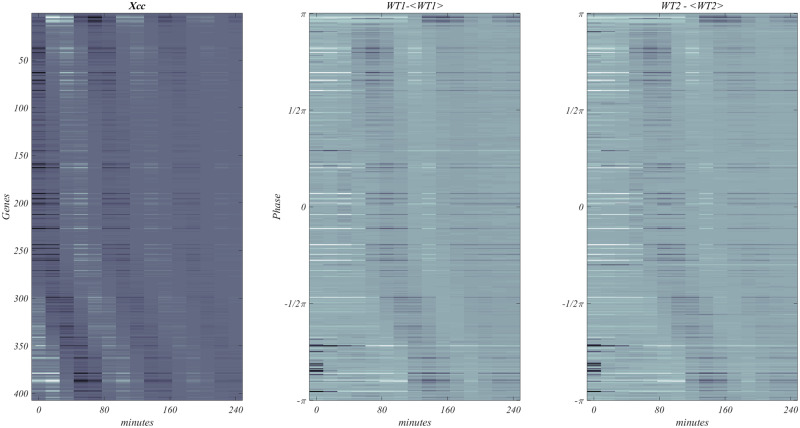
The WT400 CDC wave. Three activity images of the approximately 400 high-value *CDC* genes as represented by *X*cc, left; by the mean subtracted data from WT1, middle; by the mean subtracted data from WT2, right. All three images are free of any enhancement.

In Fig. 7 below, we show three versions of the CDC phase wave.

The first panel on the left displays the phase wave for the sampled data, without the use of trigonometric interpolation. The agreement with the left figure of Fig. 6; is evident which should remove doubt about “massaging” of data. The middle image, shows the direct application of the WT400 set to the mean subtracted version to WT1, the phase wave is clear, showing that the result is independent of the construction of $X_{\text{cc}}$. Finally, the rightmost panel, clearly shows the phase wave when WT400 is applied to WT2. WT2 which played no role in the analysis, and hence is an independent verification of WT400. Supplementary File S1 provides a full list of the credentials of WT400.

### Second proof of concept

The Spellman *et al.* [10] study employed meticulous application of Fourier methods to obtain phase information. Along with lab knowledge this produced a set of 800 genes deemed to be “cell-regulated genes,” that has 272 genes in common with WT400. The search for WT400 included consideration of set of approximately 800 genes, WT800, dropped from consideration since it produced a fainter phase wave. The intersection of this larger set with the Spellman 800 gave 390 genes.

Further comparison is furnished by tests proposed by [21]. Of these tests, that labeled “Dberg_benchmark_smallscale,” contains 113 genes is regarded as a “gold standard” of CDC genes, and has the high, 73, commonality with WT400. Another of the tests, labeled Pacifica, contains the 25 highest amplitude genes. However, based on the present criterion for “highest amplitude,” only three Pacifica genes qualify. The criterion for judging a gene to belong to the CDC order is ambiguous. Nevertheless, it seems clear that WT400 has high commonality with accepted orders. But, by this measure, there are several hundred genes WT400, that merit further examination. Supplementary File S2 shows these comparisons, and Supplementary File S3, the credentials of the present WT800.

## Discussion

A mathematically inclined reader might be surprised that a dynamical system of approximately 6000 genes, can be adequately described by a mere six-dimensional space, especially when each gene likely requires several nonlinear dynamical equations to be properly modeled [23] (It is a speculation that a more frequently sampled experiment will not change this aspect the picture.). The explanation is that these *CDC* genes are slaved to a single underdamped oscillator Equation (24).

In [10] the authors introduce the concept of co-regulated genes, and use methods largely unconnected to the present analysis. One aspect of this is the suggestion that genes of nearby phase might be co-regulated. The three Supplementary files that accompany this paper allow the reader to look into this as a phase related feature. One can imagine that the co-regulation of *CDC* genes manifests itself through a temporal process of growth and decay. As demonstrated in the Appendix, DMD would be capable of detecting such an event. For example, if experimental time sampling is performed on a minute by minute basis, it is a speculation that cell regulating genes might be uncovered through a scenario of growth and decay.

In this regard, mention should be made that comparison with Spellman *et al.* brings up a concern. In their analysis, each gene expression, g, is normalized so that

(30) $$g(t)\to \ln_{2}(g(t)/\bar{g}),\;\bar{g}={\langle g\rangle }_{t},$$

which differs from Equation (3) in two essential ways. Dividing by $\bar{g}$, sometimes referred to as “whitening” puts weak and strong gene expression on the same footing, and the log transformation is questionable. On the other hand, the relatively strong agreement between WT400 and WT800 with Spellman800, mentioned above, suggests that more is going on, and the need for further investigation.

A partial model for the lifecycle of the yeast cell can be proposed. In this regard, it is first noted that at the moment of cell division, each daughter cell has half the proper number of organelles: mitochondria, endoplasmic reticulum, Golgi apparatus, etc. The proposal is to take WT400 (or say WT800), specified by Equation (22) as controlling the CDC. A large number of remaining genes performs their role at a steady rate, without phase, so e.g. organelle density grows until the proper density is reached. A gaping hole of the model is how does the self-assembly of the cell take place. It is a speculation that some structures such as the Golgi apparatus, endoplasmic reticulum, mitochondria, etc., already present in the daughter cells, serve as seeds for their self-assembly.

Finally, as pointed out by a referee, there is potential utility of this method for discovering new circadian genes from transcriptome dynamics data, and the author would be happy to provide help to anyone who wishes pursue this possibility.

## Supplementary Material

bpaa018_Supplementary_Data

## Figures and Tables

**Figure 8:**
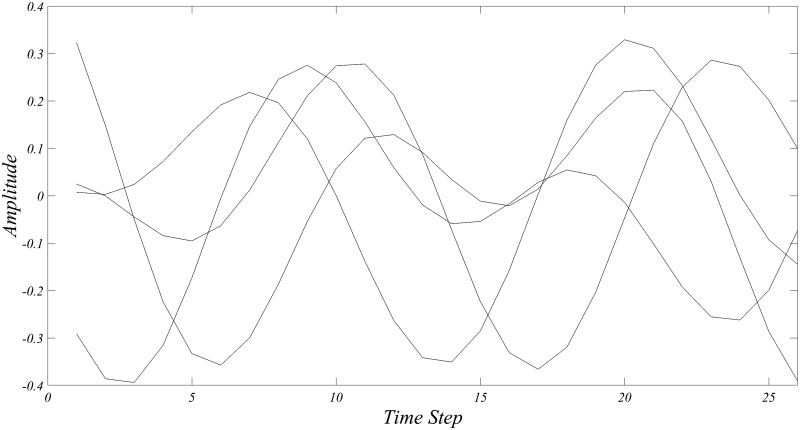
The four SVD modes of Equation (A.4).

## Appendix

**Outline of DMD**

The goal of this section is to provide guidance in carrying out DMD calculations (Readers in need of aid with this outline may contact the author for help.). To flesh out the DMD analysis, we consider a toy example. All coding will be performed in the Matlab language [24].

If the data understudy were an admixture pure sinusoids then a spectral analysis [25], would suffice and unraveling the entangled data. However, it should be evident that exponential growth and decay are playing a role, and that a more robust analysis is needed. One purpose of this appendix is to demonstrate that DMD fulfills this role.

*Toy Example*: A time course driven by many incommensurate frequencies looks to the eye as chaotic. For purposes of exposition, we will consider just two frequencies, 2 and π,

(A.1) $$T(t)=[\alpha_{1},\alpha_{2},\alpha_{3},\alpha_{4}]*{\left[\text{sin}(2t),\;\text{cos}(2t),\;\text{sin}(\pi t),\;\text{cos}(\pi t)\right]}^{†},$$

where each *α_j_* is chosen, at random, from the uniform distribution over the unit interval.,

In analogy with laboratory data *D* will be time sampled. The first consideration is the Nyquist–Shannon criterion [20], which states that a sampling frequency must be greater than twice the highest frequency latent in the data. A second requirement is that the time duration of the sample be larger than the period of the smallest frequency. From these deliberations, the sampling period P = 4.5, >2π/2, and a sampling rate dt = 0.18,<2/2π, are chosen. Thus there are 26 sample points

(A.2) t=[0,dt,2dt,….,4.5].

As a nominal case consider an ensemble of 100 presentations, yielding a data matrix, *D*, of order 100 × 26, given by

(A.3) D=C*T,

where *C* is order 100 × 4, with each element chosen at random over the interval [0, 1], and thus the matrix *D* is order 100 × 26. The first step is to get rid of “noise.” Consider the SVD of *D* which in Matlab is given by [u, s, v]=svd(D); s is the diagonal matrix of singular values and s^2^ the matrix of eigenvalues, of which *n* = 4 are significant, as should be expected. See Matlab script below.

$$\;\begin{array}{c} \begin{array}{c} \%\;\text{Noise}\;\text{removal}\;D\to \text{Do},\;N=100\\ \%\text{find}\;r,\text{the}\;\;\text{number}\;\text{of}\;\text{signals},\;4\; \end{array}\\ \text{clear}\;u\;v\;s\\ {\text{Do}=\text{USV}}^{†} \end{array}\;$$

In regard to the above script, the time dependence is contained in

(A.4) $$T={\text{SV}}^{†}\;\text{or}\;V^{†}.$$

In the Figure 8 below, we exhibit the four temporal modes of $V^{†}.$

It should be clear that the modes are entangled versions of the two input frequencies 2 and π.

As discussed earlier, the goal of DMD is to untangle the dynamics. Without loss of generality, we can restrict attention to temporal behavior. The criterion for solving Equation (14) [17, 18], is equivalent to solving

(A.5) $$\tilde{A}T1=T2,$$

where

(A.6) T1=T(1,…,N−1) & T2=T(2,…,N),

for $\tilde{A}$. A simple dynamical example is

(A.7) $$T=[e^{0dt\times z},e^{1dt\times z},e^{2dt\times z},\ldots .,e^{\mathrm{Ndt}\times z}],\;\text{with}\;\;z=\lambda +i\omega ,$$

where λ and ω are real. The period is P = 2π/ω and dt=P/N. In this case Equation (A.5) is solved by $\tilde{A}=e^{z}$, i.e. multiplication by the complex generator $e^{z}.$Observe that the complex conjugate, $T^{*},$is also a candidate solution. Since we are dealing with real quantities, and that require real results. In this spirit, we write T=C+iS,where

(A.8) $$\begin{array}{c} C=e^{\lambda \cdot t}\;\text{cos}(\omega \cdot t)\;\&\;S{=}^{\lambda \cdot t}\;\text{sin}(\omega \cdot t)\;;\;\\ t=[0,1,\ldots N]\times dt. \end{array}$$

Equation (A.5), for the real case in the same notation is solved by with an order 2 matrix generated,

(A.9) $$\left(\begin{array}{cc} e^{\lambda dt}\;\text{cos}(\omega \cdot dt) & -e^{\lambda dt}\;\text{sin}(\omega \cdot dt)\\ e^{\lambda dt}\;\text{sin}(\omega \cdot dt) & e^{\lambda dt}\;\text{cos}(\omega \cdot dt) \end{array}\right)\left[\begin{array}{c} C1\\ S1 \end{array}\right]=\left[\begin{array}{c} C2\\ S2 \end{array}\right].$$

In general, we can expect a solution for Equation (A.5) to have the form

(A.10) $$\tilde{A}=\left[\begin{array}{cccc} {\tilde{A}}_{1} & O & O & O\\ O & {\tilde{A}}_{2} & O & O\\ O & O & \ddots & O\\ O & O & O & {\tilde{A}}_{M} \end{array}\right],{\tilde{A}}_{k}=\left[\begin{array}{cc} \alpha_{k} & -\beta_{k}\\ \beta_{k} & \alpha_{k} \end{array}\right],\;O=\left[\begin{array}{cc} 0 & 0\\ 0 & 0 \end{array}\right].$$

Matrices of this form, off-diagonal skew-symmetric, are normal and have a straightforward eigen theory, can

We return to the toy problem and apply the following Matlab script:

$$\begin{array}{l} \%\text{DMD}\;\text{Script}\\ {}[u,s,v]=\text{svd}(D);U=u(:,1\text{:r});\;S=s(1\text{:r},1\text{:r});\;V=v(:,1\text{:r});\\ \text{clear}\;u\;v\;s\\ Vt=V;\;\%\\ \text{clear}\;U\;S\;V\\ V2=\text{Vt}(:,1:25);\%T2\\ V1=\text{Vt}(:,2:26);\;\%T1\\ \tilde{\text{A}}=V2*\text{pinv}(V1);\;\%\text{Moore}-\text{Penrose}\;\text{inverse}\\ \text{L}=\text{eig}(\tilde{\text{A}});\\ \text{dt}=.18;\;\%\text{time}\;\text{step}\\ \text{omega}=\text{log}(L)/\text{dt};\;\;\%\;\text{Gives}\;\;\;\Omega ,\;\text{the}\;\text{frequencies} \end{array}$$

The result of applying this script to *V* are the omega values ±i2 and ±iπ, to double precision accuracy, for any *N* > 4.

We deal specifically with *X,* as given by Equation (11), the noise-free signal made up of 5716 rows, and *N *=* *15 sampling times at intervals *dt* = 16 min, and restricted to the significant *r* (= 6) modes

(A.11) $$X=U_{r}\Lambda_{r}V_{r}^{†}=U_{r}T_{r}.$$

Henceforth the subscript *r* will be dropped. The notation of Equation (A.11) emphasizes that the dependence on gene activity and on time appears in factored form, i.e. for example, in $\sum_{k}U_{jk}T_{k}$ the first term, *U_jk_*, specifies the nature and quality with which each gene affects the kth time course, *T_k_.*

Under the notation of Equation (13), the goal of minimizing Equation (14) is achieved by solving

(A.12) $$\tilde{A}T1=T2,$$

for $\tilde{A}\;\;$ where,

(A.13) $$T=\Lambda V^{†}=[\tau_{1},\tau_{1},\ldots ,\tau_{N}],$$

And T1 and T2 are defined by Equation (A.6). formally

(A.14) $$\tilde{A}=T2\times T1^{+}.$$

with $T^{+}$ the Moore–Penrose inverse. From Equation (A.13)  *T* is the collection of the *r* time courses shown in Fig. 3, and from Equation (A.11) all gene expressions have the same time course, modified by individual amplitudes, *R*, and phases *φ*, determined by *U*.

It follows from this that for

(A.15) $$T=[{\tilde{A}}^{0}\tau_{1}\;{\tilde{A}}^{1}\tau_{1}\;\cdots {\tilde{A}}^{14}\tau_{1}\;]=W[D^{0}\;D^{1}\;D^{2}\cdots D^{14}]W^{-1}\tau_{1},$$

*X* be expressed as

(A.16) $$X=UW[D^{0},D^{1},\ldots D^{N-1}]W^{-1}\tau_{1}.$$

If

(A.17) $$\Phi =[D^{0}\;D^{1}\;D^{2}\cdots D^{14}]\times \Phi_{0},\Phi_{0}=W^{-1}\tau_{1},$$

X can be expressed in two alternate ways,

(A.18) $$\begin{array}{c} X=(UW)\Phi =U_{W}\Phi ,\;\\ \text{or}\\ X=U(W\Phi )=U\Phi_{W}. \end{array}$$

The first form, with modal form Equation (19) is the desired DMD representation in terms of disentangled modes, evolving in time through powers of *D*. The amplitude and phase of each time course is determined by *U_W_*as exhibited in Fig. 4. The second form of Equation (A.18) determines amplitude and phase from *U*, and entangles the dynamics through the product $W\Phi =\Phi_{W}$, as in the SVD decomposition, with modal decomposition Equation (11).

### Supplementary data

Supplementary data is available at *Biology Methods and Protocols* online.
